# Transcranial photobiomodulation for reducing symptoms of autism spectrum disorder and modulating brain electrophysiology in children aged 2–7: an open label study

**DOI:** 10.3389/frcha.2025.1477839

**Published:** 2025-01-29

**Authors:** Yuli Fradkin, Joaquin A. Anguera, Alexander J. Simon, Luis De Taboada, Eugenia Steingold

**Affiliations:** ^1^ Robert Wood Johnson Medical School, Rutgers University, New Brunswick, NJ, United States; ^2^ Director of Clinical Division, Neuroscape Associate Professor, Neurology and Psychiatry, Weill Institute for Neurosciences & Kavli Institute for Fundamental Neuroscience, University of California, San Francisco, CA, United States; ^3^ Interdepartmental Neuroscience Program, Yale University, New Haven, CT, United States; ^4^ JelikaLite Corp., New York, NY, United States

**Keywords:** ASD, autism spectrum disorder, photobiomodulation, transcranial photobiomodulation, EEG, delta power, pediatric neurology

## Abstract

**Background:**

Small pilot studies have indicated that transcranial photobiomodulation (tPBM) may help alleviate symptoms of neurological conditions like depression, traumatic brain injury and Autism Spectrum Disorder (ASD).

**Objective:**

To examine the effect of tPBM on the behavioral symptoms of ASD and brain electrophysiology in children aged 2–7.

**Methods:**

We conducted an open label, one-arm study with 23 participants, aged 2–7, previously diagnosed with ASD. We delivered non-invasively to all participants pulses of near-infrared light (wavelength 850 nm, pulse 40 Hz) to cortical nodes of Default Mode Network, Broca and Wernicke areas, and occipital lobe of the brain, twice weekly for 10 weeks. The tPBM was delivered using an investigational medical device designed for this purpose. Changes in ASD symptoms were measured using pre- and post-intervention scores on the Childhood Autism Rating Scale (CARS-2, 2nd Edition). We collected electroencephalogram (EEG) data after each treatment session from all children who tolerated wearing the EEG cap to monitor changes in brain activity.

**Results:**

The intervention resulted in a significant 7-point reduction in average CARS-2 scores (*t* = 10.23, *p* < .0001), along with decreased delta power and increased gamma and beta power in EEG readings. The increase in gamma power was statistically significant [*t*(14) = 2.30, *p* = 0.047]. Changes in EEG power were significantly correlated with the number of sessions (delta: *r*(192) = −0.18, *p* = .013; gamma: *r*(192) = .19, *p* = .007; beta: *r*(192) = .15, *p* = .04). Improvements in CARS-2 scores were negatively correlated with changes in delta and beta power (delta: *r*(15) = −.59, *p* = .020; beta: *r*(15) = −.54, *p* = .037). No moderate or severe side effects were reported.

**Conclusion:**

This study supports the potential of tPBM as a safe and effective treatment for ASD, and it suggests that EEG measurements may serve as a useful biomarker for future research.

**Trial Registration:**

https://clinicaltrials.gov/ct2/show/NCT04660552

## Introduction

Autism Spectrum Disorder (ASD) is a neurodevelopmental condition, usually identified within the first two years of a child's life. It impacts a person's social and communication abilities and is marked by repetitive behaviors and language challenges. ASD affects all racial and ethnic groups and significantly influences the quality of life of affected children and their caregivers, with symptom severity and type varying greatly among affected individuals. Parents of children with ASD often face increased parenting stress, mental and physical health issues, and financial strain compared to parents of children without ASD (1). ASD poses a substantial financial burden on affected families, with recent studies estimating the average per capita lifetime cost of ASD to be over $3.5 million in the United States (2). ASD is a huge societal financial burden that continues to increase with the growing prevalence of ASD in the population. The Center for Disease Control (CDC) estimated that in 2020 the prevalence of ASD in children was 1 in 36, a nearly 90% increase from the 1 in 68 prevalence reported in 2010—with a yearly burden to the US economy expected to exceed $460 billion by 2025 (3, 4).

A full understanding of the pathogenesis of ASD remains elusive (5). However, it has been established that ASD can be hereditary in some instances, and having a close family member like a cousin or sibling with ASD or another mental health disorder increases the risk (3, 4). Numerous studies suggest that environmental contaminants, such as pesticides, heavy metals, and air pollutants, might contribute to ASD development (6, 7). Additionally, maternal diet and substance use during pregnancy are identified as risk factors (8).

Several theories have been proposed to explain the etiology of ASD, including mitochondrial dysfunction (5), neuroinflammation (9–11), overproduction of reactive oxygen species (ROS), abnormal immune responses, and impaired cellular metabolism (12). Mitochondrial dysfunction is present in as many as 80% of children diagnosed with ASD and it is correlated with a variety of ASD symptoms, such as developmental and cognitive disabilities, language impairment, gastrointestinal dysfunction, and fatigue (13, 14).

Neuroinflammation, specifically activation of microglia cells for an extended period, leads to the sustained production of inflammatory mediators for longer than usual. This long-term increase in inflammatory mediators contributes to loss of synaptic connections and neuronal cell death (9–11), processes that are associated with long range neuronal underconnectivity, a phenomenon reported in many autism studies (15–18). Relatedly, there is a demonstrated correlation between mitochondrial dysfunction and neuroinflammation, in which molecules derived from damaged mitochondria activate inflammatory pathways (19).

The prevalence in ASD of both under- and over-connectivity of the default mode network (DMN) might be related to mitochondrial dysfunction and neuroinflammation (20). Several studies have correlated DMN dysfunction with conditions such as dementia, schizophrenia, and autism (21). Specifically, functional MRI studies have identified aberrant DMN connectivity in both children and adults with ASD (22).

Transcranial photobiomodulation (tPBM) therapy, a non-invasive and painless approach that leverages visible red light (600–700 nm) and/or invisible near-infrared light (NIR, 800–1,200 nm) to treat neurological disorders. tPBM has been shown to effectively restore or enhance mitochondrial function (23–25), reduce inflammation and oxidative damage (26), and potentially modulate default mode network (DMN) connectivity (27, 28). tPBM is being trialed as a therapeutic intervention for ASD with encouraging results. Ceranoglu (29) demonstrated significant reduction of symptoms in adults with high-functioning ASD (Social Responsiveness Scale, 2nd Edition) after 8 weeks of tPBM stimulation (pulsed, 850 nm) of the forebrain. Pallanti (30) reported a small study in which tPBM successfully reduced symptoms of ASD. Fradkin et al. (31) demonstrated significant reduction of symptoms of ASD in 2- to 6-year-old autistic children (CARS-2, 2nd Edition or “CARS-2”) after 8 weeks of tPBM stimulation with the investigational medical device (40 hz, 850 nm) of targeted brain regions, which included some cortical nodes of the DMN as well as some areas in pre-frontal cortex and the temporal lobe.

In this study, we administered the tPBM treatment using the investigational medical device and assessed the generalizability of tPBM as a therapeutic intervention for children aged 2–7 with ASD. We used CARS-2 to score the severity of ASD symptoms pre- post-intervention and collected EEG data of known neural signatures after each tPBM session using a high-resolution EEG device, specifically designed for young children (Ant-Neuro Eego Sports 32). We focused on clinical results, without seeking validation of the mechanism of action of PBM therapy in ASD.

## Materials and methods

### Study design

This was an 8-week open-label exploratory study designed to validate the safety and efficacy of the tPBM device designed for pediatric autistic patients.

The study protocol was approved by WCG institutional review board (IRB, 1280247) and is registered at ClinicalTrials.gov (Identifier: NCT04660552).

### Participants

Two- to seven-year-olds, inclusive of both genders and all ethnicities, with a previous diagnosis of ASD and CARS-2 scores above 28, were eligible to participate in the study. The diagnosis had to be given by a licensed professional (e.g., a developmental pediatrician) in accordance with the *Diagnostic and Statistical Manual of Mental Disorders, fifth edition* (32). These evaluations typically involved a battery of tests, including CARS-2, Autism Diagnostic Observation Schedule (ADOS), Temperament and Atypical Behavior Scale (TABS), and Autism Diagnostic Interview-Revised (ADI-R).

Detailed inclusion and exclusion criteria are presented in Table 1.

**Table 1:** Inclusion and exclusion criteria.

Inclusion criteria	
1.	Male or female participants between 2 and 7 years of age (inclusive).
2.	Previously diagnosed with ASD by a licensed professional.
3.	Participants may be receiving any behavioral intervention therapy (e.g., ABA) during the treatment.
4.	Parents of participants must understand the nature of the study.
Exclusion criteria	
1.	Participant is experiencing severe self-injurious behavior or severe aggressive behavior to self or others (within the past 7 days).
2.	Participant has been diagnosed with another psychiatric or neurological disorder (e.g., epilepsy) or has a history of seizures or have exhibited symptoms of major psychiatric disorders within the last 30 days.
3.	Participant has an unstable medical condition that requires clinical attention.
4.	Participant has a significant skin condition at the procedure sites.
5.	Participant has an implant of any kind in the head.
6.	Participant is receiving medication on a regular basis.
7.	Any use of light-activated drugs.
8.	Participant is a member of investigators' immediate family.

Recruitment was conducted through Applied Behavioral Analysis agencies and specialized schools, specific to the ASD population.

Informed consent was obtained for each treatment session.

### Setting

Treatments were applied and clinical data collected in an IRB approved location.

### Investigational product and treatment procedure

**Figure 1:**
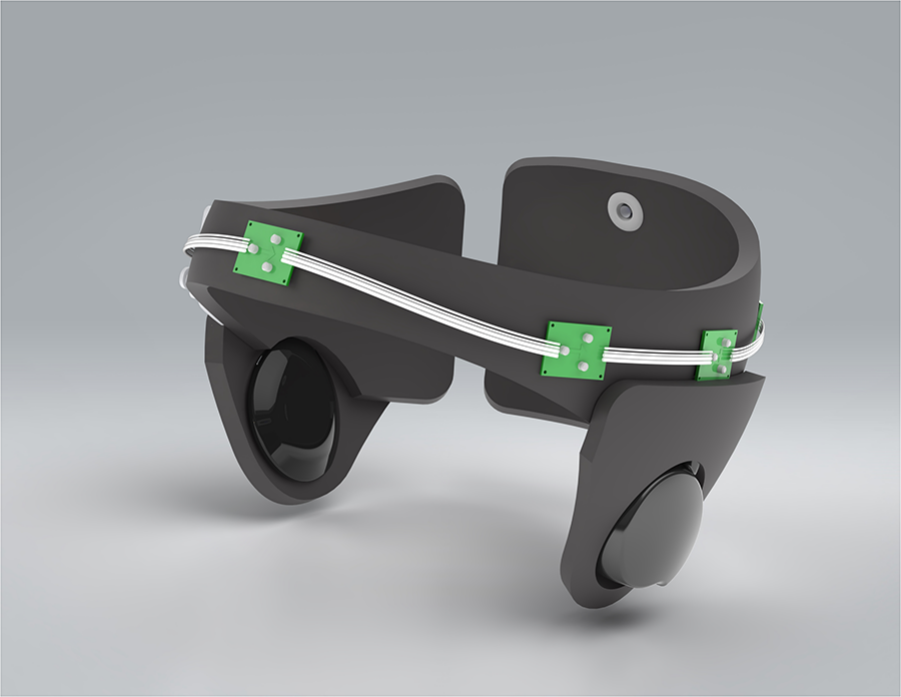
Image of investigational medical device.

The device under investigation was: a wireless, light-weight device specifically designed for young children with ASD to remain mobile (see Figure 1). The device is Bluetooth controlled and provides transcranial delivery of pulsed NIR light (40 Hz, 850 nm), via 6 LEDs (150 mW maximum optical power, each) to targeted brain areas (which included amongst others, cortical nodes of DMN, Broca and Wernicke areas, occipital lobe and prefrontal cortex).

Following a two-week titration protocol (described below), each participant underwent treatment twice a week for 8 weeks. Each treatment session was followed by 15 min of EEG data collection from participants that tolerated wearing the EEG cap.

*Titration protocol:* Each participant started with a 2 min tPBM treatment. Participants who tolerated the treatment well received incrementally larger doses (2 min) in each of 5 subsequent sessions, for a maximum dose of 12 min.

## Measures

### Demographics

Age, gender, race, and ethnicity were collected by staff before intervention.

### Primary outcome: CARS-2: childhood autism rating scale

To evaluate the effect of tPBM on ASD symptoms, pre- and post-intervention assessments of CARS-2 were conducted on all participants by the last author, a licensed psychologist experienced in the use of CARS-2 in clinical practice. To minimize the potential bias present in open-label studies, the evaluator did not see the pre-treatment CARS-2 scores during the post-treatment CARS-2 evaluations.

CARS-2 is a validated clinical rating scale designed for use by trained clinicians to assess ASD based on direct observation of the child (33). It comprises 15 items, each corresponding to core domains affected by ASD. Total scores on the scale range from 15 to 60. Interpretations of scores are categorized as follows: scores below 30 indicate the non-autistic range; scores from 30 to 36.5 indicate mild to moderate autism; scores from 37 to 60 indicate severe autism.

To evaluate the impact of tPBM on brain electrophysiology, trained staff members proficient in operating the EEG devices conducted EEG data collection sessions lasting 15 min after each tPBM session. EEG data was collected utilizing the eego™ sports 32, a dry-electrode, 32-channel EEG device specifically engineered for use in young children (ANT Neuro GmbH, Germany). Data collection was limited to children comfortable with wearing the EEG cap.

EEG is used for brain electrophysiology, providing valuable insights into the neural dynamics associated with ASD, aiding diagnostic information, and informing early intervention strategies (34–37).

### Analysis

#### Primary outcome: CARS-2

Pre- and post-intervention CARS-2 scores were analyzed and compared using a paired samples *t*-test. Changes in mean CARS-2 scores were considered significant at *p* < 0.05.

#### Secondary outcome: EEG

EEG data was preprocessed using the EEGLAB software (38). Noisy channels identified upon initial visual inspection were removed from the data and interpolated using a spherical spline interpolation, using the average signal of the surrounding channels to reconstruct the data in the removed channel. The data were detrended, and a low-pass filter at 50 Hz was applied to remove high-frequency noise. Ocular correction was performed by removing segments of data containing eye blinks and lateral eye movements. The data were then re-referenced to the average signal of all channels. Event-related spectral perturbation (ERSP) power in delta (1–4 Hz), theta (4–7 Hz), alpha (8–12 Hz), beta (15–30 Hz), and gamma (30–50 Hz) frequency bands were resolved using a fast Fourier transform (FFT).

Here we interrogated the recorded ERSP power by: (1) using a correlation-based analysis involving scaled time as in (31) using a traditional pre-post intervention analysis. For the first approach, we evaluated global power by collapsing the ERSP power signal across all electrodes. To assess changes in spectral power over scaled time, Pearson correlations were performed to test the relationship between spectral power in each band collected at each treatment session for each participant. Given that participants had different number of treatment sessions, to evaluate change in this signal, *only participants who had at least 3 treatment sessions were included in the analysis*. For the second approach, we directly investigated spectral power pre- and post-treatment using each individual's first and final treatment sessions by paired *t*-test analysis. Given the small sample size of this study, each test functioned following a bootstrapping procedure where 1,000 samples were taken to achieve 95% confidence interval threshold. Results were significant at *p* < 0.05.

## Results

### Study participants (sample characteristics)

Thirty-one children aged 2–7 years were screened to ensure they met all inclusion and none of the exclusion criteria. Most participants received their diagnosis during the 24 months evaluation period provided through the New York State Department of Health Early Intervention program.

Twenty-five children were initially enrolled. Three dropped out due to travel limitations, leaving twenty-two participants who completed the study.

The mean age of the participants was 4.9 years old, with a standard deviation (SD) of 1.46 years. Four (4) were 7 years old. Nineteen were males. 86.36% of the participants were White, 4.55% were Black, 4.55% were Central Asian, and 4.55% were South Asians.

Baseline demographics data are presented in Table 2.

**Table 2:** Baseline demographics data.

No. of patients (*n*)	22
Age, years, mean (SD)	4.95 (1.46)
Sex, *n* (%)	
Male	19 (83.36)
Female	3 (13.64)
Ethnicity, *n* (%)	
White	19 (86.36)
Asian/South Asian	2 (9.09)
Black	1 (4.55)
Verbal status *n* (%)	
Verbal	16 (72.73%)
Non-verbal	6 (27.27%)
Baseline CARS-2 score, mean (SD)	36.45 (5.55)

### Clinical results

#### Treatment procedure and dosing

All twenty-two participants that completed the study tolerated the treatment well and received up-to 12 min treatments twice a week for 8 weeks. Four participants experienced headaches. Seventeen experienced hyperactivity at least once during the study, according to their parents. Fifteen participants tolerated at least three 15 min EEG recording sessions.

#### Primary outcome: CARS-2

After the intervention, a significant reduction in CARS-2 scores was observed, with a mean decrease of 7 points (*t* = 10.23, *p* < .0001). The pre-intervention CARS-2 scores had a mean of 36.5 (SD = 5.6, *n* = 22), while post-intervention scores averaged 29.7 (SD = 5.5).

Detailed CARS-2 scores for each participant, along with the overall mean (M) and standard deviation (SD), are presented in Table 3. Participant's pre- post-intervention score changes by CARS-2 sub-scale are tabulated in Table 4.

**Table 3:** CARS-2 scores, mean (SD).

No. of patients (n)	22
Mean (SD)	
Before	36.45 (5.55)
After	29.68 (5.47)
Mean change (95% CI)	6.77 (5.36–8.19) *p* < .0001

**Table 4:** Patient's CARS-2 Sub-scale change: post-intervention score Minus baseline score.

CARS-2 SUB-scale	Patient																					
	1	2	3	4	5	6	7	8	9	10	11	12	13	14	15	16	17	18	19	20	21	22
Relating to People	−1	0	0	−0.5	−0.5	−0.5	−0.5	−1	−0.5	−0.5	−0.5	−1	0	−0.5	0	−0.5	−0.5	−0.5	0	−1	−1	0
Imitation; Social-Emotional Understanding	−0.5	−0.5	0	−0.5	−0.5	−0.5	−1	0	−0.5	−0.5	−1.5	0	0	−0.5	−0.5	−0.5	−0.5	−0.5	−0.5	−1.5	−0.5	−0.5
Emotional Response; Emotional Expression and Regulation of Emotions	0.5	−0.5	−1	0	−0.5	0.5	−0.5	0	−1	−0.5	−1	0	−1	−0.5	−1	−0.5	−1.5	−0.5	−0.5	−0.5	−1	0
Body Use	−0.5	−0.5	−0.5	0	0	−1	0	−1.5	−0.5	0	−0.5	−0.5	0	−0.5	−1	−0.5	−1.5	0	−1.5	−0.5	−0.5	−0.5
Object Use; Object Use in Play	0	−0.5	−0.5	0.5	−0.5	−1	−0.5	1	0	0	−0.5	−0.5	0	−0.5	0	−1	−1.5	−0.5	−0.5	−2	−1.5	0
Adaptation to Change; Adaptation to Change/Restricted Interests	1	−1	−0.5	0.5	0	−0.5	−0.5	−0.5	−0.5	−0.5	0	0.5	0	0	−1	−1	−1	−0.5	0	−0.5	0	−0.5
Visual Response	−0.5	−1.5	−0.5	0	−1	−0.5	0	−1	−1	−0.5	−0.5	−1	1	0	−0.5	0.5	0	−0.5	−0.5	−1	0	−0.5
Listening Response	−1	−1	−0.5	−0.5	0.5	−1	−0.5	−0.5	−0.5	0	−1	0	0.5	−1	−0.5	−1	0	−1.5	−0.5	−1	−0.5	0
Taste, Smell, & Touch Response and Use	−1	−1.5	−0.5	0	−2	0	−0.5	0	−0.5	−0.5	−1	0	1	−1.5	−1	0	−2	−1.5	−0.5	−1	−0.5	−1
Fear or Nervousness/Fear or Anxiety	−0.5	−1	0	0	−0.5	0.5	0	−1	−1	0	−0.5	0.5	0	−0.5	0	−0.5	−0.5	0	0	−1	−0.5	−0.5
Verbal Communication	−1	−0.5	0	−1	−0.5	0.5	−0.5	0	−1	0	−1	−0.5	0	−0.5	0.5	0	−0.5	−1	−0.5	−0.5	−1	−0.5
Nonverbal Communication	−0.5	−1	0	0	−1	−0.5	0	−0.5	−0.5	−0.5	−1	0	−0.5	−0.5	0	−0.5	−1	−1	0	−0.5	−1	−0.5
Activity Level/Thinking/Cognitive Integration Skills	−0.5	−0.5	−0.5	−0.5	−1	−0.5	0	0.5	−0.5	−0.5	0.5	0.5	0	−0.5	−1	−0.5	−1.5	−0.5	0	−0.5	−1	−0.5
Level and Consistency of Intellectual Response	−1	−0.5	0	0	−0.5	0.5	0	−1	−0.5	0	0	−0.5	−0.5	−0.5	0	0	−0.5	−1	−1	−0.5	−0.5	0
General Impressions	−1	0	0	−0.5	0.5	0	−0.5	−0.5	−1	−0.5	−0.5	0	0	0	0.5	0	−1	−0.5	−1	−0.5	0	0

Although the very small sub-group sample sizes make conclusions speculative, a *post-hoc* analysis of the data revealed no significant differences in the reduction of mean CARS-2 scores between participants aged 2–6, participants aged 7.

*The observed 7-point change in mean CARS-2 scores before and after the intervention aligns with the results from the* (31)*, where the difference in mean CARS-2 score change between the two groups was 7.23 (95% CI: 2.357–12.107, p* *=* *0.011)*.

The CARS-2 scores result for the (31) study are reproduced in Table 5.

**Table 5:** CARS-2 scores, mean (SD) (31),.

	Active group	Sham group
No. of patients (n)	16	14
Mean (SD)		
Before	43.5 (5.7)	40.6 (7.2)
After	33.7 (5.0)	38.0 (8.4)
Mean change (95% CI)	9.875 (7.541–12.109)	2.643 (1.973–7.258)
Difference between groups (95% CI)	7.23 (2.357–12.107) *p* < 0.01	

#### Secondary outcome: EEG

**Figure 2:**
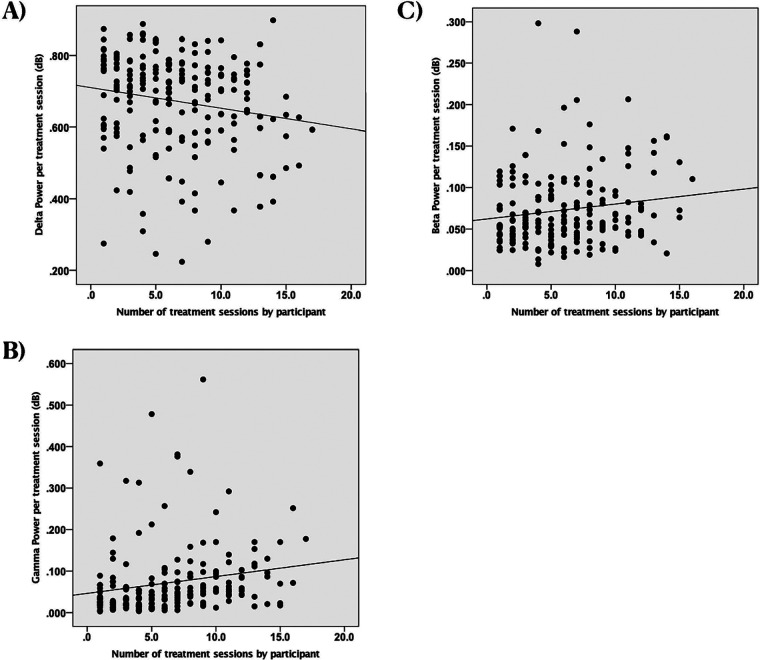
Scatterplots of spectral power in different frequency bands vs. the number of treatment sessions with EEG recordings by participant, dB, decibels (unitless measure of power amplitude). **(A)** Change in delta power. Each dot represents an individual observation. The line is the regression line. *X* axis: scaled time. *Y* axis: power of delta. **(B)** Change in gamma power. Each dot represents an individual observation. The line is the regression line. *X* axis: scaled time. *Y* axis: power of gamma. **(C)** Change in beta power. Each dot represents an individual observation. The line is the regression line. *X* axis: scaled time. *Y* axis: power of beta.

In our initial ERSP analysis, we found a notable negative correlation between the number of treatment sessions and delta ERSP power (*r*(192) = −0.18, *p* = 0.013; see Figure 2A). This suggests that delta power decreased as the number of treatment sessions increased. Additionally, we observed significant positive correlations between treatment sessions and both gamma and beta power (gamma: *r*(192) = 0.19, *p* = 0.007, see Figure 2B; beta: *r*(192) = 0.15, *p* = 0.04, see Figure 2C), indicating that these neural signals strengthened over time. No significant correlations were found in the other frequency bands (theta: *r*(192) = 0.03, *p* = 0.72; alpha: *r*(192) = 0.07, *p* = 0.34).

**Figure 3:**
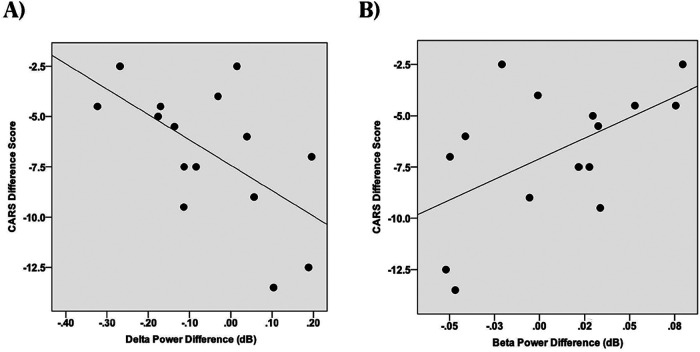
Scatterplots of change in spectral power (last session minus first session) in different frequency bands vs. the Childhood Autism Rating Scales (CARS-2), dB, decibels (unitless measure of power amplitude). **(A)** CARS-2, EEG delta power correlation. **(B)** CARS-2, EEG beta power correlation.

We also identified significant negative correlations between improvements in CARS-2 scores (a measure of symptom severity) and changes in both delta power (*r*(15) = −0.59, *p* = 0.020; see Figure 3A) and beta power (*r*(15) = −0.54, *p* = 0.037; see Figure 3B). However, no significant associations were found between CARS-2 scores and changes in gamma or theta power (gamma: *r*(15) = −0.43, *p* = 0.11; theta: *r*(15) = 0.11, *p* = 0.70).

Our second ERSP analysis, which directly compared ERSP power between the first and final treatment sessions, showed a significant pre- post-treatment increase in gamma power (*t*(14) = 2.30, *p* = 0.047). There were no significant differences in any of the other frequency bands.

## Discussion

The present open-label one arm study investigated the effect of tPBM (delivered by the investigational medical device) on the symptoms of ASD and brain electrophysiology.

We evaluated changes in ASD symptoms using the CARS-2, comparing scores before and after the tPBM intervention. To assess brain activity, we analyzed EEG data collected after each tPBM session using Ant-Neuro EEG cap. After the eight-week tPBM treatment, we observed a significant reduction in ASD symptoms and notable changes in brain activity. These results closely mirrored those of the (31), which were achieved using the same investigational medical device^.^

The observed 7-point improvement in mean CARS-2 scores suggests clinically significant changes as (39) established in their seminal work, that the change of 4.5 points is clinically significant. Specific CARS-2 sub-scale changes (Table 4) indicate meaningful progress in several core areas affected by autism, such as communication, social interaction, and sociability. However, assessing a clinically significant difference in ASD symptoms using CARS-2 is challenging. Many notable improvements, such as holding hands while crossing the street or using the bathroom independently, were not captured by CARS-2 due to its lack of sensitivity to these changes, despite the significant enhancement they brought to family quality of life. Future research should incorporate additional scales, such as the Aberrant Behavior Checklist (40) and the Social Responsiveness Scale (41).

The study replicated the decline in delta power and its correlation with improved CARS-2 scores previously reported in findings from (31). We replicated the significant reduction in delta power over time which correlated with post-treatment decline in CARS-2. The current study also found significant correlations between EEG gamma and beta power with the duration of the intervention, as well as changes in gamma power before and after treatment. Higher power of Gamma waves is associated with improved memory and cognition (42–44).

The observed changes in brain activity were associated with improvements in ASD symptoms and shifts toward more typical brainwave patterns.

Although speculative, the observed changes in delta brainwave power may indicate reduction of neuroinflammation, which is known to affect ASD symptoms (13, 14, 45). Frohlich et al. (45) argued that delta waves in wakeful states are often associated with various neurological conditions including TBI, chronic hemorrhage, microglial activation, and inflammation. Furthermore, the presence of delta waves in the wakeful state was associated with the locations of future seizures (46). If this is the case, these findings align with prior research on non-pharmacological treatments that have shown similar brain activity changes correlate with improved performance on untrained tasks (18, 47, 48). On a related note, research by (46) suggests that increased delta power during wakefulness may signal a heightened risk for seizures, while Weiss (49) found a link between delta waves and epileptiform activity in adults. These findings imply that tPBM could be a promising treatment option for individuals with ASD who also experience seizures, highlighting the need for more research.

These findings should be interpreted with caution. Studies comparing EEG patterns in children with ASD to neurotypical children have produced mixed results (50–52), and we only observed changes in gamma power after the intervention. Despite this, the results suggest that EEG pattern changes could be a valuable tool for assessing treatment effectiveness, personalizing therapy, and guiding future research.

## Limitations

Several limitations should be acknowledged when interpreting these findings. First, the sample's heterogeneity, limited sample size, lack of a direct control group, and relatively wide age range could confound the results with natural developmental changes. Second, some children were unable to tolerate the EEG cap after each tPBM session. Third, although the CARS-2 is an FDA-required outcome measure for ASD trials, it may not optimally capture the full range of symptom improvements, especially those that substantially impact family life but are not well-reflected in the scale. Additionally, the study did not include follow-up assessments (CARS-2 or EEG) months later to determine the persistence or transience of the treatment effects.

Given these limitations, the results should be interpreted with caution. They should be considered only within the specific population and conditions under which the study was conducted. Broad generalizations or extrapolations beyond this context may lead to inaccurate conclusions or misinterpretations.

## Conclusions

This open-label study adds significant support to using tPBM as a safe and effective intervention for reducing the core symptoms of ASD. Nevertheless, larger-scale studies with more rigorous designs are necessary to validate and extend these results. Future research should specifically examine the impact of light-dosing variables—such as pulsing frequency—on treatment outcomes; assess long-term efficacy and potential side effects; investigate drug interactions; and analyze changes in specific symptoms by evaluating each CARS-2 subscale individually. Additionally, studies should evaluate the effects on both the child's and parents' quality of life. It is also important for future research to consider factors like gender, functional impairment, verbal status, and concomitant therapies, as well as the high comorbidity of ASD with other neurological and psychiatric disorders—including attention deficit hyperactivity disorder (ADHD), obsessive-compulsive disorder (OCD), anxiety, and cognitive disabilities. Moreover, given that a significant percentage of children diagnosed with ASD develop seizures later in life, further studies employing EEG are warranted.

## Data Availability

The raw data supporting the conclusions of this article will be made available by the authors, without undue reservation.
